# ADAR2-Mediated Editing of miR-214 and miR-122 Precursor and Antisense RNA Transcripts in Liver Cancers

**DOI:** 10.1371/journal.pone.0081922

**Published:** 2013-12-27

**Authors:** Wan-Hsin Liu, Chao-Hung Chen, Kun-Huei Yeh, Chiao-Ling Li, Yi-Jinn Wu, Ding-Shinn Chen, Pei-Jer Chen, Shiou-Hwei Yeh

**Affiliations:** 1 Department of Microbiology, National Taiwan University College of Medicine, Taipei, Taiwan; 2 National Taiwan University Center for Genomic Medicine, National Taiwan University College of Medicine, Taipei, Taiwan; 3 Department of Oncology, National Taiwan University College of Medicine, Taipei, Taiwan; 4 Department of Internal Medicine, National Taiwan University College of Medicine, Taipei, Taiwan; 5 Graduate Institute of Clinical Medicine, National Taiwan University College of Medicine, Taipei, Taiwan; 6 Department of Laboratory Medicine, National Taiwan University Hospital, Taipei, Taiwan; University of Hong Kong, Hong Kong

## Abstract

A growing list of microRNAs (miRNAs) show aberrant expression patterns in hepatocellular carcinoma (HCC), but the regulatory mechanisms largely remain unclear. RNA editing catalyzed by members of the adenosine deaminase acting on the RNA (ADAR) family could target the miRNA precursors and affect the biogenesis process. Therefore, we investigate whether RNA editing could be one mechanism contributing to the deregulation of specific miRNAs in HCC. By overexpression of individual ADARs in hepatoma cells, RNA editing on the precursors of 16 miRNAs frequently deregulated in HCC was screened by a sensitive high-resolution melting platform. The results identified RNA precursors of miR-214 and miR-122 as potential targets edited by ADAR2. A subset of HCC showing elevated ADAR2 verified the major editings identified in ARAR2 overexpressed hepatoma cells, either with A-to-I or U-to-C changes. The unusual U-to-C editing at specific residues was demonstrated as being attributed to the A-to-I editing on the RNA transcripts complementary to the pri-miRNAs. The editing event caused a decrease of the RNA transcript complementary to pri-miR-214, which led to the decrease of pri-miR-214 and miR-214 and resulted in the increased protein level of its novel target gene Rab15. In conclusion, the current study discovered ADAR2-mediated editing of the complementary antisense transcripts as a novel mechanism for regulating the biogenesis of specific miRNAs during hepatocarcinogenesis.

## Introduction

MicroRNAs (miRNAs) are endogenous noncoding RNAs identified as posttranscriptional regulators of gene expression. Increasing numbers of miRNAs were frequently found to be deregulated in HCC, and some have participated in the carcinogenic process through targeting of specific cellular genes involved in tumor proliferation, apoptosis, and metastasis [1], [2].

Various mechanisms have been reported contributing to the aberrant expression of miRNAs in tumors. In addition to the genomic or epigenetic changes, defects of the miRNA biogenesis process were also considered involved in the dysregulation, either transcriptionally by specific transcription factors or posttranscriptionally by processing factors [3]. Increasing number of factors participating in the posttranscriptional biogenesis steps have been identified, including the general factors for all miRNAs (such as Drosha, DGCR8, and Dicer) [4] and the specific factors for a subset of miRNAs (such as P53, TRBP2, KSRP, p68/p72, Lin28B, and others) [5], [6]. In addition to these factors, it has been considered that RNA editing could be another mechanism for posttranscriptionally regulating the miRNA level in tumors.

RNA editing results in a change of RNA sequence, which is different from the one encoded by the genome and contributes to the diversity of protein products [7]. In humans, this event is mediated by ADAR enzymes, including ADAR1 and ADAR2, which catalyze the deamination of adenosine to inosine (A-to-I) in double-stranded RNA [8]. Two ADAR1 isoforms by alternative promoter usage were identified that encode ADAR1S and ADAR1L [7], [8].

In addition to the protein coding genes, the ADARs could also target the miRNA precursors, either the pri- or pre-miRNAs, which contain the stem-loop structures as favorable substrates for the editing machinery. Aided by high-throughput sequencing analysis, it has been estimated that ∼20% of human pri-miRNAs are edited [9]. Such editing events have the potential to affect the function of miRNAs, including the stability of the precursors, the processing efficacy during biogenesis, or even the target gene selection [10], [11]. Such a possibility has already been well demonstrated in several specific miRNAs. For example, pri-miR-142 was found edited by ADAR1 and ADAR2 *in vitro*, which led to the suppression of its processing by Drosha. Another example came from the editing of pri-miR-151 by ADAR1, which inhibits the pre- to mature-miR-151 processing by blocking its cleavage by Dicer [12].

In the present study, we tried to investigate whether the RNA editing by ADARs occurs for regulating the processing of HCC-related miRNAs. As a pilot study, we expect the results could help understand if the RNA editing events contribute to the deregulated expression of specific miRNAs in hepatocarcinogenesis.

## Materials and Methods

### Plasmid Constructs

The cDNA clones for human ADAR1L and ADAR2 were purchased from Mammalian Gene Collection (MGC, NIH, USA), and the ADAR1S was subcloned from ADAR1L clone. These cDNA fragments were changed into pCMV.SPORT6 vector and then proceeded further to clone into the lentiviral vector of pLenti6/V5-DEST by using Gateway Technology™ System (Invitrogen Life Technologies Inc.).

The pLKO.1-siLuc and pLKO-1-siGFP constructs driven by U6 promoter was obtained from the RNAi Core Facility in Academia Sinica, Taiwan (Taipei, Taiwan). In addition, several pLKO.1 vector-based siRNA plasmids were constructed by insertion of the specific siRNA sequence into the pLKO.1-siLuc vector at the restriction sites of AgeI and EcoRI. The sequences for each siRNA inserts against the sense and antisense transcripts of miR-214 and miR-122 were listed as followed. siRNA for miR-214: 5′-CCTTTGCTCATAGACAGATAC-3′; siRNA for AS-miR-214: 5′-GTATCTGTCTATGAGCAAAGG-3′; siRNA for miR-122: 5′-CCTTCGTGGCTACAGAGTTTCC-3′, siRNA for AS-miR-122: 5′-TGCCAAGACATTTATCGAGGG -3′. The details for preparation of lentiviruses expressing ADARs or specific siRNAs were as described previously [13].

To construct the plasmids expressing either the sense or the complementary antisense transcripts of pri-miR214 and pri-miR122 in culture cells, we have cloned the PCR products amplified from genomic DNA of Huh-7 cells into the pCMV.SPORT6 vector. The primer sets were designed to comprise the precursor miRNA with ∼200 bases extending to both 5′ and 3′ flanking sequence of each miRNA. The mutations of specific residues in these constructs, including A-34G and A-27G for pri-AS-miR-214 and A-7G for pri-miR-122, were introduced by site-directed mutagenesis using the QuickChange II Site-Directed Mutagenesis Kit (Stratagene, Cedar Creek, TX), by following the manufacture's instruction.

The reporter construct pGL3-Rab15-3′UTR, containing 3′UTR of Rab15 gene, was constructed by inserting the DNA fragment covering nt. 1∼714 of 3′UTR of Rab15 gene (nt. 1 of 3′UTR as the first nucleotide after the stop codon of Rab15, at nt. position 709 of Rab15 transcript, NM_198686) into pGL3-Promoter vector (Promega, Madison, WI). The DNA insert was amplified by PCR from the genomic DNA of Huh-7 cells with primers of 5′-GGTCCGTCTAGACAGAGCTGGTGCTGCAGGCCCAT-3′ and 5′-GAAGGCTCTAGACGAGGCAGGGTGGAG GGTTCTTC-3′.

### High-resolution Melting (HRM) Analysis

HRM is a powerful tool developed for sensitive sequence variant screening, utilizing the DNA property of heat separation in the presence of saturation fluorescence dyes, such as the High Resolution Melting Dye (Roche Diagnostics Applied Science, Mannheim, Germany) used in this study. The heteroduplex DNA fragments can be distinguished from the homozygous ones by showing a different-shaped melting curve [14]. This enables the detection of single base pair changes in DNA fragments up to 400 bp by melting curve analysis. HRM analysis is thus suitable for our identifying the nucleotide changes caused by editing events of pre-miRNAs (∼100 bps in average).

The primers were designed for each miRNA using the LightCycler Probe Design Software, with the sequences summarized in the Table S1. The lengths of each amplicon were ∼200 bps, which covering the entire pre-miRNAs with at least 50 bps extending to 5′ and 3’-ends. The PCR amplification and HRM analysis was conducted by the LightCycler® 480 (Roche Diagnostics Applied Science), with details as described [15].

The PCR products containing the heteroduplex fragments were identified by showing the relative signal difference of the melting curves compared with those amplified by the genomic DNA of Huh-7 cells (as the standard without editing), using the Gene Scanning Software (Roche Diagnostics Applied Science). To avoid the false positive results caused by the assay variations, we have set the cut-off values for each amplicon by analyzing the relative signal difference among PCR products from 12 repeats of PCR reaction with Hu-7 genomic DNA as template, which was <3.5 in all the cases. Therefore, the cut-off values for the relative signal difference were set as 5 in our analysis.

### Study Subjects

The liver tissues from 20 HBV related male HCC patients collected in the Taiwan Liver Cancer Network (TLCN) were included in this study. The DNA and RNA from the paired tumorous and the adjacent nontumorous liver tissues from each patient were used for the reverse transcription and PCR reactions. Then the PCR product was processed for the sequencing analysis, aiming to identify the residues with putative editing events. The resected surgical specimens were quickly kept in liquid nitrogen until the DNA and RNA extraction. The Institutional Review Board of National Taiwan University Hospital approved the use of these archived tissues, and all patients gave their written informed consents before enrollment.

### Quantification of Pri-miRNA and Mature miRNAs

Total RNA was isolated from the cells with Trizol reagent (Rezol C&T, PROtech, Taipei, Taiwan) and then processed for the reverse transcription using the Superscript III One-Step RT-PCR System (SuperScript® III First-Strand Synthesis System, Invitrogen, Carlsbad, CA), for the subsequent quantification of pri-miRNA. The mixtures for reverse transcription reaction contained 1 µg of RNA, 10 µM of specific primer, 5′-ATATCAGATGAACCTTCTTGCTC-3′ for pri-miR122 and 5′-GGTTGTAGCTCTTGGTGTAGATG-3 for pri-miR-214, with the reaction details as described [13].

The qPCR was conducted by LightCycler FastStart DNA Master SYBR Green I Kit (Roche, Mannheim, Germany) in LightCycler PCR machine with details as described [13]. The primers used for specific amplification of pri-miR214 and pri-miR122 are listed as followed. Pri-miR-214: 5′-GTATCTGTCTATGAGCAAAGGAAACC-3′ and 5′-GGTGTAGATGCTATGGTGTGAGGGC-3′; pri-miR-122: 5′-ACCTTCGTGGCTACAGAGTTTCC-3′ and 5′-TGCCAAGACATTTATCGAGGGAAGG-3′.

Quantification of mature miR-214 and miR-122 was performed using the TaqMan miRNA Assay Kit (TaqMan® MicroRNA Reverse Transcription Kit, Applied Biosystems, Foster City, CA) according to the manufacturer's instruction. U6 RNA was used as an internal control for determining the relative miRNA expression level with details as described [13].

### Strand Specific qRT–PCR Analysis

The strand specific qRT–PCR for the primary transcript of pri-miR-122 and pri-miR-214, and also their complementary antisense transcripts, were conducted by following the protocol as described by Ho *et al* [16]. In brief, 2.5 ug of RNA extracted from cells or tissues was processed for the reverse transcription (RT) reaction by strand specific primer; and then the strand specific RT product was used for the qPCR analysis. The strand specific primers designed for pri-miR-122 and pri-miR-214 are miR-122S: 5′-ACCTTCGTGGCTACAGAGTTTCC-3′ and miR-214S: 5′-GTCTGCCTGTCTACACTTGCTG-3′; and those designed for the complementary transcripts are miR-122AS: 5′-TGCCAAGACATTTATCGAGGGAAGG-3′ and miR-214AS: 5′-AGGCTGGGTTGTCATGTGACTG-3′. The same set of primers for strand specific RT reaction was used for the subsequent qPCR analysis with details as described [13].

### TA Cloning and Sequencing Analysis

The RT–PCR products from the lenti-ADAR2-infected cells were purified by gel extraction and cloned into the yT&A vector (Yeastern Biotech Co. Ltd., Taipei, Taiwan) by TA cloning. The positive clones containing inserts were processed for sequencing analysis to detect the nucleotide changes, in comparison with the sequence from the control samples without ADAR2 overexpression.

### Cell Culture, Transfection, Luciferase Reporter Assay, and Western Blot Analysis

Two hepatoma cell lines, HepG2 and Huh-7 cells, were purchased from BCRC (Bioresource Collection and Research Center, Taiwan, http://www.bcrc.firdi.org.tw). The genotype of these two cell lines has been authenticated by BCRC using AmpFI STR Identifiler PCR amplification kit of Applied Biosystems for extensive STR PCR DNA analysis, with the profiles identical with the ATCC data. The details for cell maintenance, transfection, luciferase reporter assay, and the Western blot analysis were as described previously [17]. The antibodies used for the Western blot analysis included anti-ADAR2, anti-GUTL1, anti-IGF-1R, and anti-Rab15 (Santa Cruz Biotechnology, CA), anti-ADAR1 (Abnova, Taoyuan), and anti-β-actin (Sigma, St. Louis, MO).

## Results

### Editing of miR-214 and miR-122 Precursors in Huh-7 Cells by Overexpression of ADAR2

**Figure 1:**
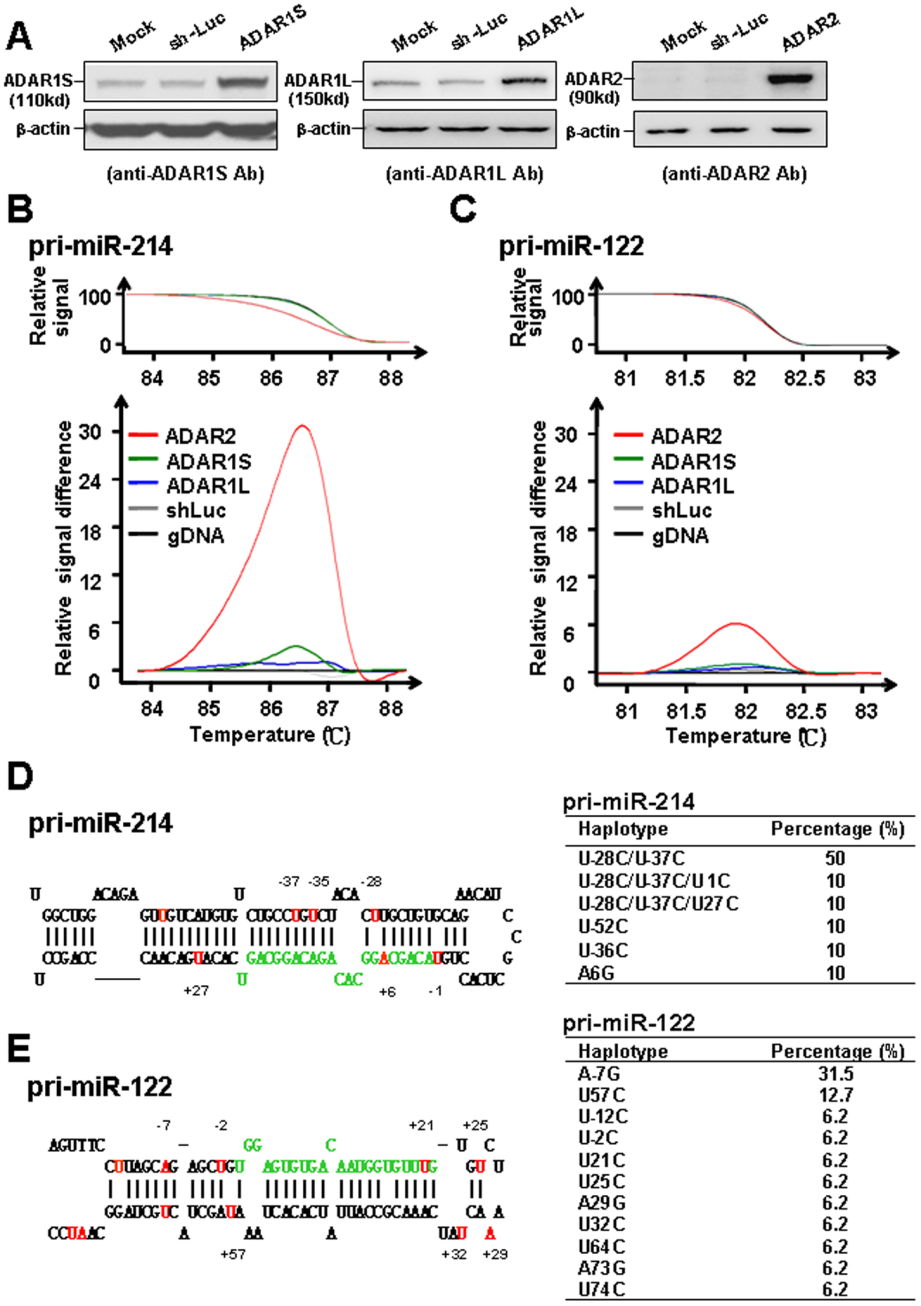
Specific editing of the RNA transcripts related with pri-miR-214 and pri-miR-122 in Huh-7 cells infected with lenti-ADAR2 lentiviruses. (**A**) The cells were uninfected (mock), infected with lenti-sh-luc (negative control), or infected with the lenti-ADAR1S, ADAR1L, and ADAR2 individually. The cell lysates were processed for Western blotting by probing with Abs as indicated at the bottom of each panel. The HRM results for miR-214 (**B**) and miR-122 (**C**). The raw data for the relative signals are shown in the upper panel; and the difference of the relative signal by comparing with the “no editing” standard are shown in the lower panel. The stem loop structures for pre-miR-214 (**D**) and pre-miR-122 (**E**) are illustrated schematically, with the nucleotides corresponding to mature miRs labeled with green. The positions of the nucleotides changed by overexpression of ADAR2 are marked in red, with the numbers showing their positions relative to the first nucleotide of mature miRs (as position no. 1). The summary results of the nucleotide changes in precursors of these two miRNAs are shown at the right panel, by sequencing of 100 clones from TA cloning of the RT–PCR products amplified by the RNA from lenti-ADAR2 infected cells.

To examine if RNA editing contributes to the aberrant expression of specific miRNAs in hepatocarcinogenesis, 16 miRNAs reproducibly reported as deregulated in HCC (Table S1) were chosen for our testing if they were substrates edited by ADARs. We took the approach by overexpressing individual lenti-ADARs in Huh-7 cells, including ADAR1S, ADAR1L, and ADAR2, and then examined if RNA editing caused any nucleotide changes in the precursors of these miRNAs. The increased protein expression of individual ADARs was first verified by Western blot analysis (Fig. 1A). The RNA was extracted from cells overexpressing individual ADARs for RT–PCR and subsequent HRM analysis.

The PCR product amplified by the genomic DNA of Huh-7 cells, which apparently is not the target of ADARs, served as the “no editing” standard for the HRM analysis. By comparing with such a standard, the relative signal scores for each of the 16 miRNAs from cells infected with individual lenti-ADARs were calculated, with the results summarized in Table S2. The aberrant melting curves, showing the relative signal score >5, were identified in the RT–PCR products amplified by pri-miR-214 and pri-miR-122 precursor RNAs extracted from lenti-ADAR2 infected cells (Fig. 1B for miR-214 and Fig. 1C for miR-122). The results indicated that ADAR2 overexpression can introduce some mismatches in the transcripts of pri-miR-214 and pri-miR-122, suggesting the precursors could be the substrates for ADAR2.

To identify further the specific residues in the primary transcripts of these two miRNAs edited by ADAR2, the RT–PCR products from the lenti-ADAR2-infected cells were processed for TA-cloning, and the subsequent sequencing analysis was conducted in 100 clones for each pri-miRNA. Several recurrent nucleotide changes were identified to be different from the sequence of samples without ADAR2 overexpression. Fig. 1D (for pri-miR-214) and Fig. 1E (for pri-miR-122) schematically illustrated their positions relative to the mature miRs, which also summarized the percentage of these nucleotide changes in the sequenced clones. The most frequent changes occurred with the haplotype of U–28C/U–37C for pri-miR-214 (in ∼50% of clones) and with the haplotype of A–7G for pri-miR-122 (in ∼30% of clones).

### ADAR2 Elevation Correlated with the Nucleotide Changes at Specific Residues of Pri-miRNAs in HCC Tissues

**Figure 2:**
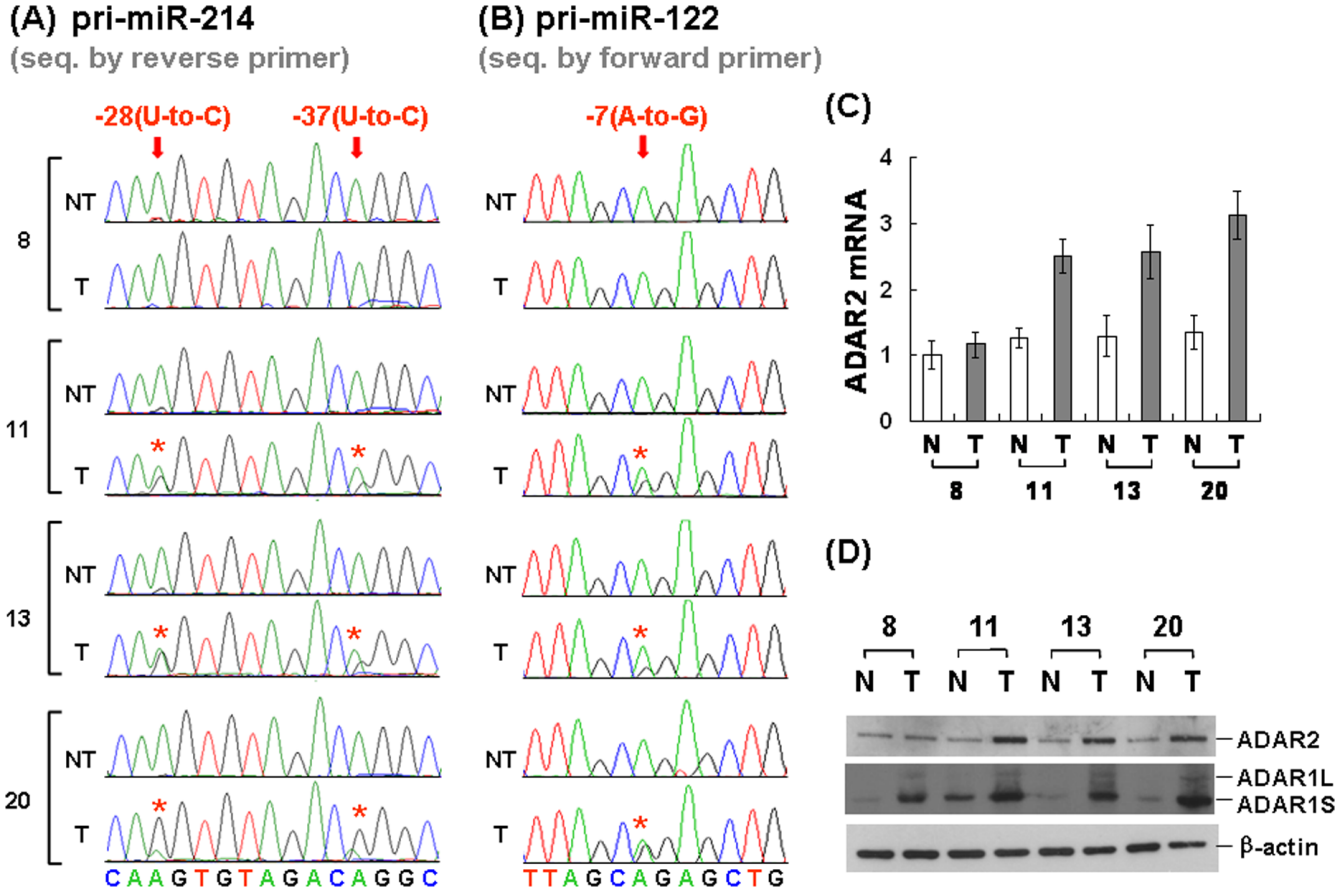
The ADAR2-mediated editing at specific nucleotide residues in the RNA transcripts related with pri-miR-214 and pri-miR-122 also occurred in the clinical HCC specimens. The representative sequencing results for miR-214 (**A**) and miR-122 (**B**), with the RT–PCR products from four pairs of HCC tissues as indicated. The nucleotide residues with changes in the tumorous (T) tissues, in comparison with those in the corresponding nontumorous (NT) tissues, are marked with red arrows. The positions relative to that of the mature miRNAs are indicated above the arrows. (**C**) The relative expression levels of ADAR2 mRNA in these HCC tissues were determined by qPCR analysis, with the quantitative results shown as relative to the level of the NT part of patient no. 8. (**D**) The protein expression of ADAR2 and ADAR1 in these four pairs of HCC tissues was examined by Western blotting.

Next, we examined if the ADAR2-meditated recurrent nucleotide changes identified in Huh-7 cells also occurred in clinical specimens. The cDNA from 20 pairs of HCCs and their corresponding adjacent nontumorous tissues were analyzed by direct sequencing of their RT-PCR product from the primary transcripts of pri-miR-122 and pri-miR-214. Three HCC tissues showed clear nucleotide changes (Fig. 2, patient no. 11, 13, and 20), with U-to-C changes at −37 and −28 of pri-miR-214 (Fig. 2A, revealed as A-to-G changes by sequencing with the reverse primer), and an A-to-G change at −7 of pri-miR-122 (Fig. 2B, revealed as A-to-G changes by sequencing with the forward primer). Such changes did not occur on the PCR product from genomic DNA of the same HCC tissues (data not shown). Interestingly, these nucleotide changes were the same as the major ones identified in the ADAR2-overexpressed Huh-7 cells (Fig. 1D, 1E), supporting these editing events could also occur in clinical specimens. It was noteworthy that these specific nucleotide changes mainly occurred in the tumors but little in the adjacent nontumorous tissues of these patients (Fig. 2A and 2B, NT vs. T), suggesting this as an event preferentially occur in tumors.

Since our *in vitro* studies showed that these nucleotide changes could be caused by ADAR2 overexpression, we examined if ADAR2 was elevated in HCC showing specific nucleotide changes. The expression levels for RNA and protein of ADAR2 in these three pairs of HCCs were examined by qPCR and Western blot, with one pair of HCCs without any nucleotide changes as the control (patient no. 8). Both the RNA and the protein levels of ADAR2 were elevated significantly in the HCC tissues of these three patients, but not in the control case (Fig. 2C, 2D). The results suggested that ADAR2-mediated RNA editing events could also occur in a subset of HCC, which is well associated with the increased expression of ADAR2.

### Editing of RNA Transcripts Complementary to the RNA Transcripts of Pri-miR-214 and Pri-miR-122

It is well documented that RNA editing by ADAR2 mainly causes the A-to-I/(G) changes [8]; however most of the nucleotide changes we identified for pri-miR-214 and pri-miR-122 precursors in ADAR2-overexpressed Huh-7 cells were U-to-C changes, except for one at position −7 of miR-122 (Fig. 1D and 1E). Since such unusual U-to-C changes are complementary to the expected A-to-G changes, we proposed the possibility that the ADAR2-mediated A-to-I editing could occur at the RNA transcripts complementary to these two pri-miRNAs.

**Figure 3:**
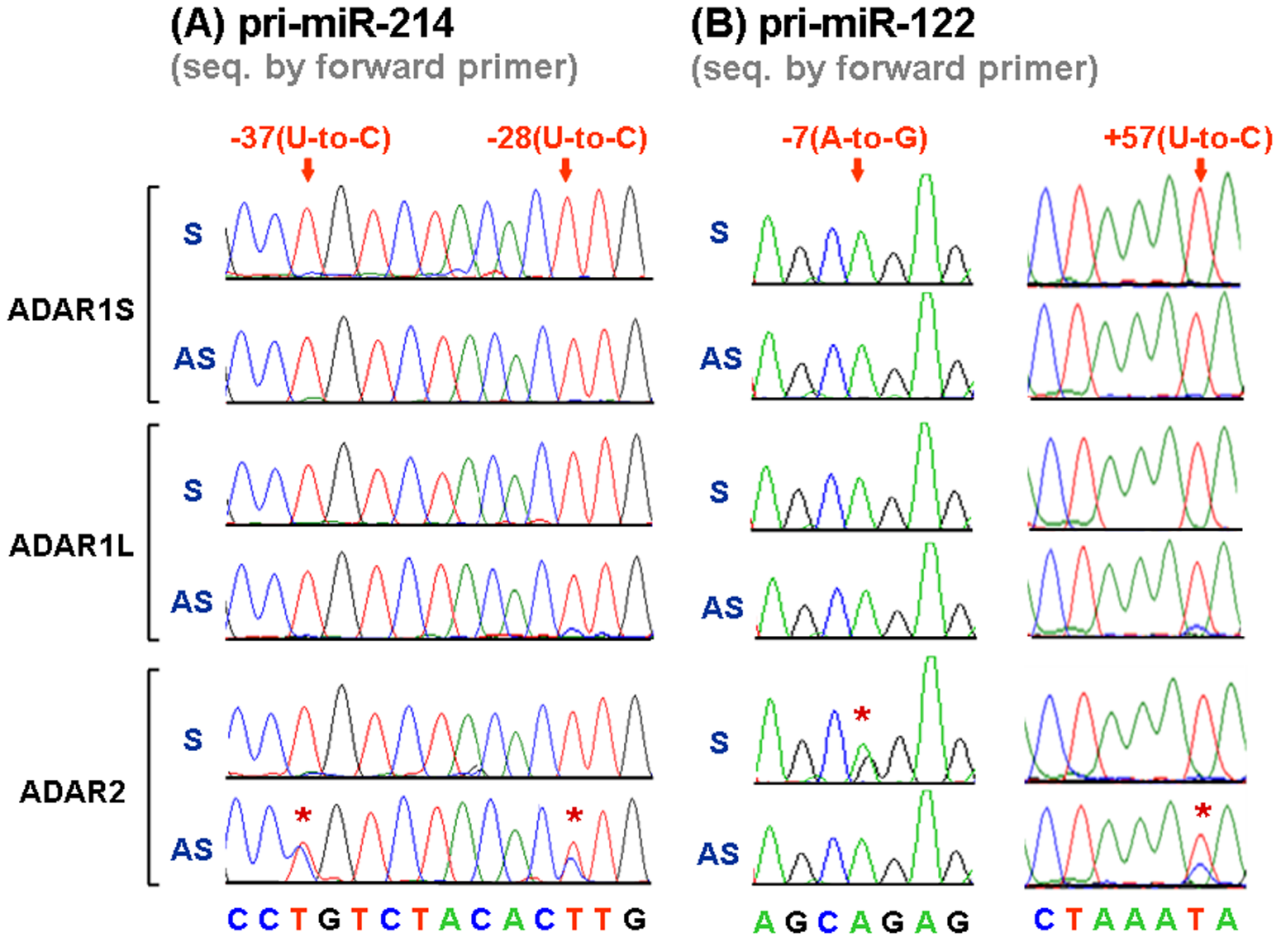
ADAR2 edits the specific nucleotide residues at the precursor and complementary antisense RNA transcripts of pri-miR-214 and pri-miR-122. The Huh-7 cells were transfected with the plasmid constructs expressing either the sense (**S**) or the complementary antisense (**AS**) transcripts of pri-miR-214 (**A**) and pri-miR-122 (**B**), and then infected with lenti-ADARs as indicated. The RNA was extracted from each cell preparation for RT–PCR and subsequent direct sequencing analysis. The results shown here are representative, focusing on the regions covering the residues identified as the major editing sites (as revealed in Figure 2), including −37 and −28 for pri-miR-214 and −7 and +57 for pri-miR-122 (marked with arrows). The nucleotide residues showing editing events are marked with red asterisks.

To test this possibility, we took the approach by overexpressing either the pri-miR-214/pri-miR-122 (sense) or their complementary RNA transcripts (antisense) in Huh-7 cells individually, in combination with overexpression of ADAR2. Such a design can help distinguish the editing events on either the pri-miRNAs or the complementary antisense transcripts. Under the overexpression of either transcripts for both pri-miRNAs (>50-fold, revealed by qPCR analysis), we found that ADAR2 could induce U-to-C changes only in the cells overexpressing the complementary antisense transcripts (Fig. 3A, −28 and −37 for AS of miR-214; Fig. 3B, +57 for AS of miR-122).In contrast, the A-to-G changes were identified only in the cells overexpressing the pri-miR-122 (Fig. 3B, −7 for S of miR-122). This supported that the observed U-to-C changes could be due to the ADAR2-mediated A-to-I editing in the RNA transcripts complementary to both pri-miRNAs. These changes did not occur in the cells infected with lenti-ADAR1S or lenti-ADAR1L, again supporting these editing events to be mediated by ADAR2.

**Figure 4:**
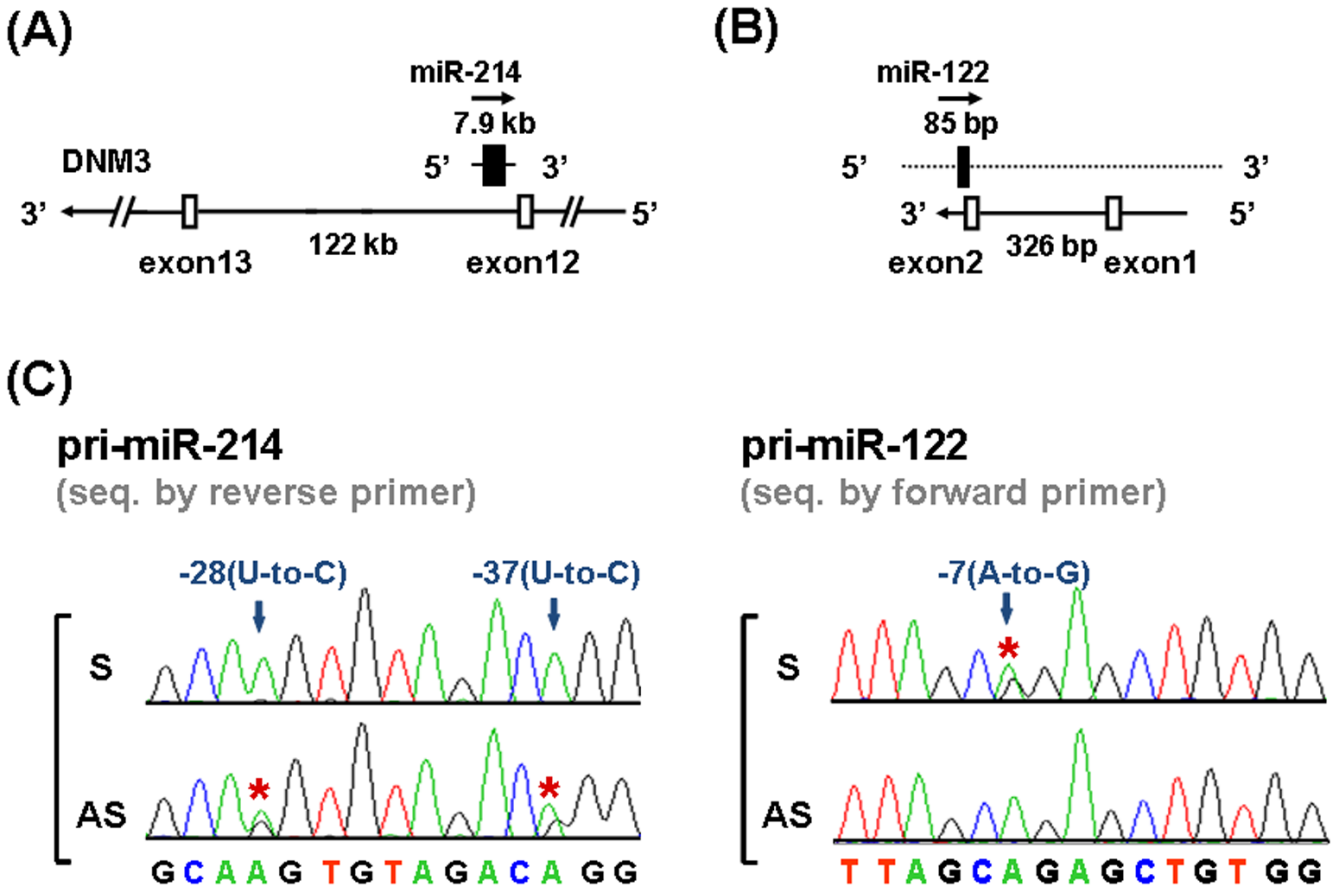
Antisense transcripts exist for pri-miR-214 and pri-miR-122, with A-to-I/(G) RNA editing at specific nucleotide residues. Schematic illustration of the gene structures for the RNA transcripts complementary to (**A**) pri-miR-214 and (**B**) pri-miR-122. (**C**) The representative results for sequencing of the strand-specific RT–PCR products from HCC with putative editing events in the sense (**S**) and antisense (**AS**) transcripts of pri-miR-214 and pri-miR-122, focusing on the regions covering the sites with potential editing events with the editing marked with red asterisks.

Since the general RT-PCR and sequencing analysis we used above cannot distinguish the editing events at sense or antisense transcripts. We thus tried to verify the specific A-to-I editing at antisense transcripts of pri-miR-214 by strand specific RT-PCR and sequencing analysis. RNA extracted from the three HCC with elevated ADAR2 (Fig. 2, patients 11, 13, and 20) were processed for the strand specific RT-PCR, to amplify the primary transcripts of these two pri-miRNAs and also their complementary antisense transcripts, for sequencing analysis. Of note, in neuroblastoma cells, Loebel et al. have already reported that the gene encoding miR-214 is located within a noncoding 7.9-kb transcript complementary to the intronic region of dynamin-3 (DNM3), between exon 12 and 13 of this gene (Fig. 4A) [18]. Aided by the two databases, GeneMark and GENSCAN, a short transcript complementary to miR-122 has been predicted (Fig. 4B).

The sequencing results showed that the editing events mainly occurred at the transcripts complementary to pri-miR-214 but not at the sense transcripts in these HCC samples (Fig. 4C, left panel, −28 and −37 of pri-miR-214). It supported that the ADAR2-mediated U-to-C changes could be attributed to A-to-I editing on the complementary transcripts of pri-miR-214 in clinical specimens. In contrast, the editing event at sites with an A-to-G pattern mainly occurred at the sense transcript in these HCC (Fig. 4C, right panel, −7 of pri-miR-122).

### ADAR2-mediated Editing on the RNA Transcript Complementary to Pri-miR-214 Functionally Affects The Target Gene of miR-214

**Figure 5:**
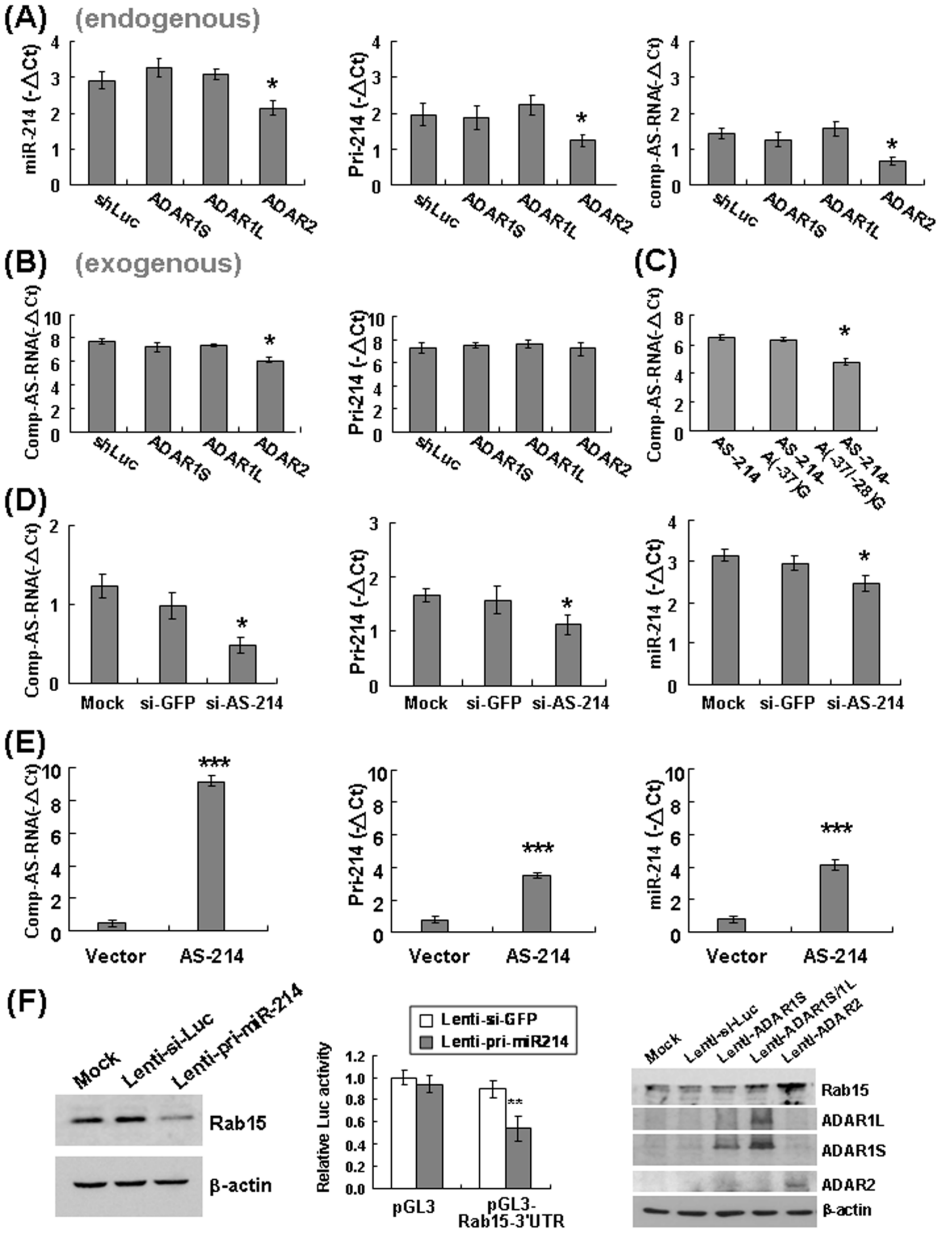
ADAR2-mediated editing on the RNA transcript complementary to pri-miR-214 decreases the levels of pri-miR-214 and the miR-214. (**A**) The RNA extracted from Huh-7 cells infected with individual lenti-ADARs as indicated was used for assessment of the levels of **endogenous** miR-214 (left), pri-miR-214 (middle), and complementary antisense transcript of miR-214 (comp-AS-RNA) (right). (**B**) The individual lenti-ADARs were infected to Huh-7 cells, which were already transfected with the plasmids **exogenously** expressing either the pri-miR-214 or the complementary antisense transcript (comp-AS-RNA) as indicated. The RNA was extracted for quantification of the levels of both transcripts accordingly. (**C**) The Huh-7 cells were transfected with the plasmid expressing the wild-type comp-AS-RNA, or the ones introducing either the single mutation as A(−37)G or double mutations as A(−37/−28)G. The levels of antisense transcript were then determined by qRT–PCR. (**D**) The Huh-7 cells were not infected (mock), infected with control lenti-si-GFP (si-GFP), or infected with lenti-si-AS-214, which targets the RNA transcript complementary to pri-miR-214, for evaluating the effects on the expression levels of comp-AS-RNA (left), pri-miR-214 (middle), and miR-214 (right). (**E**) The Huh-7 cells were transfected with the control vector or the plasmid expressing the comp-AS-RNA, for evaluating the effect on the levels of comp-AS-RNA (left), pri-miR-214 (middle), and mature miR-214 (right). (**F**) The protein lysates extracted from Huh-7 cells uninfected or infected with lenti-si-Luc or lenti-pri-miR214 were analyzed by Western blotting analysis (left). The Huh-7 cells transfected with pGL3-vector or pGL3-Rab15-3′UTR reporter constructs were infected with lenti-si-GFP or lenti-pri-miR214. The effect on reporter activity was illustrated relative to that of pGL3-vector-transfected cells infected with lenti-si-GFP (middle). The protein lysates extracted from Huh-7 cells either uninfected or infected with various lentiviruses as described were processed for Western blotting, probing with antibodies against Rab15, ADAR1, ADAR2, and β-actin (right).

The next issue is to examine the functional effect of the ADAR2-mediated editing on the biogenesis of these two miRNAs. To first focus on miR-214, the effect of ADAR2 overexpression on miR-214 as well as its precursors was analyzed in Huh-7 cells, with the cells overexpressing ADAR1S and ADAR1L included as controls. The endogenous miR-214 was decreased significantly by overexpression of ADAR2 but not by ADAR1S and ADAR1L (Fig. 5A, left panel). Both pri-miR-214 and the complementary antisense(AS) RNA transcripts (endogenous) were also decreased by overexpression of ADAR2 (Fig. 5A, middle and right panels).

To further examine the editing effects on each transcript individually, the pri-miR-214 and complementary antisense transcripts were overexpressed in Huh-7 cells separately, in combination with individual ADARs. Only the complementary RNA transcript was decreased by ADAR2 (Fig. 5B, left panel). Introduction of the A-to-G changes at positions −28 and −37 of the complementary transcript decreased its RNA level, similar to that caused by overexpression of ADAR2 (Fig. 5C). It thus demonstrated that ADAR2 could cause a reduction of the RNA transcript complementary to pri-miR-214, through the A-to-I editing at its specific residues of −28 and −37 positions.

As we noted, there is no direct effect of ADAR2 on the overexpressed pri-miR-214 (Fig. 5B, right panel, lane 4), but which did cause a decrease of endogenous pri-miR-214 (Fig. 5A, middle panel, lane 4). It raised the possibility that the ADAR2 editing caused a decrease of the complementary transcript, which in turn decreased the RNA transcript of pri-miR-214. To address this, we knocked down the RNA transcript complementary to pri-miR-214 in Huh-7 cells (Fig. 5D, left panel), which led to a decrease of pri-miR-214 (Fig. 5D, middle panel). A positive regulatory effect by the complementary transcript on the level of pri-miR-214 was thus implicated. In line with this, overexpression of complementary antisense RNA increased both the pri-miR-214 and mature miR-214 (Fig. 5E).

To examine further the functional effect of the ADAR2-mediated editing event on the target gene of miR-214, we tried first to identify its putative target gene in hepatocytes. The PicTar, TargetScan, and miRanda algorithms pointed out Rab15, a member of the RAS oncogene family, as a putative miR-214 target gene. To test this, we overexpressed miR-214 in Huh-7 cells by infection with lenti-pri-miR-214 lentiviruses, which resulted in a decrease of the level of Rab15 protein (Fig. 5F, left panel). Infection of lenti-pri-miR-214 could also repress the pGL3-Rab15-3′UTR luciferase reporter activity (Fig. 5F, middle panel), supporting that miR-214 could repress the expression of Rab15 through targeting its 3′UTR. Moreover, overexpression of ADAR2, but not ADAR1S and ADAR1L, caused an increase of Rab15 (Fig. 5F, right panel), suggesting that the ADAR2-mediated editing event has functional effect on the target gene of miR-214.

### The RNA Transcript Complementary to Pri-miR-122 Regulates the Expression Level of miR-122

**Figure 6:**
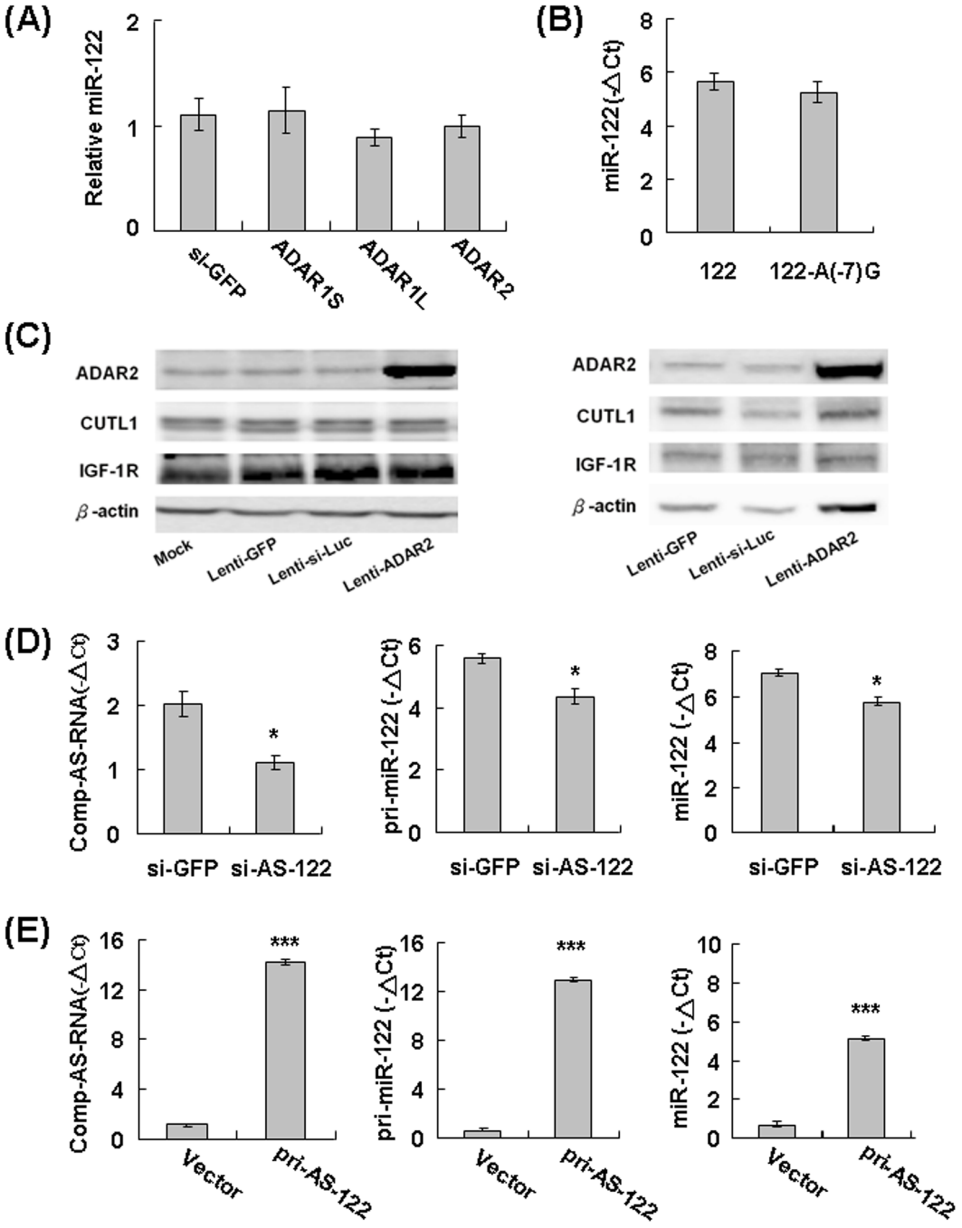
The functional influence of the RNA transcript complementary to pri-miR-122 for the expression levels of pri-miR-122 and miR-122. (**A**) RNA extracted from Huh-7 cells infected with various lenti-ADARs was prepared for assessment of the levels of **endogenous** miR-122, with that of si-GFP infected cells (set as 1). (**B**) RNA extracted from HepG2 cells transfected with either the wild-type or the mutant pri-miR-122 containing A-to-G at position −7 was processed for qRT–PCR. (**C**) The protein lysates extracted from Huh-7 (left) and HepG2 (right) cells infected with various lentiviruses were processed for the Western blotting analysis. (**D**) RNA extracted from Huh-7 cells infected with either lenti-si-GFP or lenti-si-AS-122, which targets the RNA transcript complementary to pri-miR-122, was processed for qRT–PCR for the antisense transcript (comp-AS-RNA) (left), the pri-miR-122 (middle), and miR-122 (right). (**E**) The HepG2 cells transfected with the control vector or the plasmid construct expressing comp-AS-RNA were processed for qRT–PCR, for examining the levels comp-AS-RNA (left), pri-miR-122 (middle), and miR-122 (right). The results were shown as the average of three experiments (mean ± SD), and the *P* values were calculated by *t* test (*P<0.05; ***P<0.001).

A similar analysis for miR-214 was also applied to address the effect of ADAR2-mediated editing for miR-122. First, the effect of individual ADARs on mature miR-122 was evaluated in HepG2 cells, but it did not show significant effects (Fig. 6A). Introduction of A-to-G mutation at the −7 position of the pri-miR-122 transcript, the major edited site, did not cause significant changes in the level of miR-122 (Fig. 6B). Therefore, the ADAR2-mediated editing on pri-miR-122 transcript did not affect its biogenesis process. The effect of ADAR2 on two well-characterized target genes of miR-122, CUTL1 and IGF-1R [19], [20], was also analyzed, but did not show significant changes (Fig. 6C).

Another interesting issue to be addressed concerns the effect of the RNA transcript complementary to pri-miR-122. This complementary antisense transcript has been either knocked down in Huh-7 cells (with relatively higher levels of endogenous pri-miR-122) or overexpressed in HepG2 cells (with relatively lower levels of endogenous pri-miR-122) for evaluating the resulting effects on the pri-miR-122 and miR-122. Knockdown of the complementary antisense transcript (Fig. 6D, left panel) significantly decreased both the pri-miR-122 and miR-122 (Fig. 6D, middle and right panels). Consistent with this, increase of the complementary antisense RNA transcript (Fig. 6E, left panel) induced the elevation of pri-miR-122 and miR-122 (Fig. 6E, middle and right panels). Therefore, the results suggested a positive role for the RNA transcript complementary to pri-miR-122 in regulating the level of pri-miR-122 and miR-122.

## Discussion

Aiming to investigate if ADAR-mediated RNA editing is a mechanism contributing to the aberrant expression of miRNAs in HCC, the current study identified the involvement of ADAR2 in regulating the biogenesis of miR-214 and miR-122. It is the first time that these two miRNAs reported as being regulated by RNA editing. Both miRNAs are decreased in ∼70% of HCCs [1], [2], [13]. The contribution of miR-122 in HCC has been well documented, targeting to regulate both the host genes and the viral life cycle of HBV and HCV [21]. Increasing evidence also suggested the critical role of miR-214 in hepatocarcinogenesis, through regulating the proliferation, invasiveness, and angiogenesis activities [22], [23]. Delineating the regulatory mechanisms for their decrease in HCC will provide new insights for either classification or treatment of HCC through targeting to these two miRNAs.

The specific residues edited by ADAR2 in the RNA transcripts related with these two miRNAs were not only identified in Huh-7 cells but also in human HCC with increased ADAR2 expression, supporting these editing events also occurred in a subset of HCC. However, most major editing events identified were the U-to-C pattern, which is contradictory to the known A-to-I pattern catalyzed by ADAR2. In fact, the U-to-C editing events have been reported previously in the precursors of several miRNAs [24]. Since all the known ADARs only show the activity for catalyzing A-to-I changes [7], [8], it has long been speculated that such an unusual type of U-to-C editing might be catalyzed by some other editing enzymes yet to be identified [24]. However, in our study, it is clear that the overexpression of ADAR2 can mediate such U-to-C editing. We thus proposed the possibility that such ADAR2-mediated A-to-I editing could occur at the RNA transcripts complementary to pri-miR-214 and pri-miR-122.

We noted that in most previous studies, the general RT-PCR reactions amplified both the sense and the antisense transcripts at the same time, which cannot distinguish the editing events at either sense or antisense transcript by the subsequent sequencing analysis. It might be the reason misleading to report the U-to-C editing events in specific pri-miRNA transcripts. In the current study, either the pri-miRNA (sense) or the complementary (antisense) RNA transcript of pri-miR-122 and pri-miR-214 was overexpressed in the hepatoma cells for individually examining the editing events on either transcript. The results well demonstrated that the residues with U-to-C changes identified by RT-PCR/sequencing analysis to be actually the A-to-I editing at the RNA transcript complementary to these two pri-miRNAs. This observation has been further validated in the ADAR2 overexpressed clinical specimens by strand-specific PCR analysis. Our results thus provided a possible explanation for these unusual U-to-C editing events previously identified in many pri-miRNAs, which is worthy to be investigated further.

It was noteworthy that a recent publication by Chan *et al.* identified an imbalance of ADARs in HCC, showing decreased ADAR2 and elevated ADAR1 in HCC, which might associate with increase risk of liver cirrhosis and poor prognosis [25]. In analysis of 20 pairs of HCC samples, we only found 3 HCC with elevated ADAR2 and enhanced editing. In most cases, the expression of ADAR2 is very low both in the non-tumorous and the tumorous tissues. However, we did identify ∼60% of HCC with elevated expression of ADAR1, which is consistent with the results reported by Chan *et al.* However, none of the cases with ADAR1 elevation only showed the editing events at miR-214 and miR-122 related transcripts identified in the current study, suggesting these editing events are not attributed by overexpressed ADAR1.

The functional influence of these ADAR2-mediated editing events has been addressed in the current study. For miR-214, we found that ADAR2 editing caused a decrease of the complementary antisense transcript, which in turn led to the reduction of pri-miR-214 and a decrease of mature miR-214. The biological significance of this regulation has been demonstrated by the resulting effect on the elevation of the target gene of Rab15, which for the first time has been identified as a new target of miR-214. Rab15 is a member of the Ras oncogene family, with function involved in retinoic-acid-induced differentiation and receptor recycling process [26], [27], but its role in hepatocarcinogenesis warrants further investigation.

The current study provides a mechanism for the decrease of miR-214 in a subset of HCC with elevated expression of ADAR2. However, the mechanism underlying the reduction of the complementary antisense transcript by editing at specific residues of positions −28 and −37 is still not clear yet. One possible mechanism is that the edited transcripts will become more susceptible to degradation by specific ribonucleases, such as Tudor–SN, with higher affinity for the inosine-containing dsRNAs [28]. A similar possibility has been demonstrated previously in the case of miR-142 [11], [28]. For miR-122, the major editing event at the −7 position of pri-miR-122 does not seem to affect the level of pri-miR-122 and miR-122 or the protein levels of target genes. However, the presence of a novel RNA transcript complementary to pri-miR-122 with an ability to regulate the level of pri-miR-122 and miR-122 raises a new mechanism for regulating this important miRNA in hepatocytes.

The complementary antisense transcripts exist widely for ∼70% of human genes [29], of great interest to demonstrate their functional role in carcinogenesis. Mahmoudi *et al.* recently identified a complementary antisense transcript for the p53 gene, designated as Wrap53, which could help stabilize the sense transcript of p53 mRNA. This contributed to the increase of the mRNA and protein levels of p53 as one mechanism for p53 induction upon DNA damage [30]. In addition to this example for the protein coding genes, our current results demonstrated that the RNA transcripts complementary to specific microRNAs could also positively regulate the levels of the corresponding RNA transcripts of pri-miRNAs, as a new mechanism for the deregulation of miRNAs in carcinogenesis. The results of the current pilot study will form the basis for future genomewide comprehensive analysis to reveal the whole set of miRNAs regulated by specific ADARs in HCC and other cancers.

## Supporting Information

Table S1 The primer sets used for amplification of the precursors of 16 HCC related miRNAs for HRM analysis.
(DOC)

Table S2 The relative signal scores for the HRM analysis of the precursors of 16 HCC related miRNAs from Huh-7 cells infected with specific lenti-ADARs.
(DOCX)
