# Supplementary material for: ADAR2-Mediated Editing of miR-214 and miR-122 Precursor and Antisense RNA Transcripts in Liver Cancers
Source: PLoS One. 2013 Dec 27;8(12):e81922. doi: 10.1371/journal.pone.0081922 (PMC3873926; doi:10.1371/journal.pone.0081922)
Supplement: Table S1 — The primer sets used for amplification of the precursors of 16 HCC related miRNAs for HRM analysis. (DOC) [file pone.0081922.s001.doc]

**Table S1. The primer sets used for amplification of the precursors of 16 HCC related miRNAs** for HRM analysis.

| **Specific miRNAs** | **Primer sequences** | **Reference** |
| --- | --- | --- |
| let-7c | forward 5'-GCTGACTGAAGATATGATAAGG-3'  reverse 5'-ATGACACATTACCTTCTTGC-3' | [1] |
| miR-18a | forward 5'-TGAAGGCACTTGTAGCATTA-3'  reverse 5'-ACCATCAGTTTTGCATAGATTT-3' | [2] |
| miR-21 | forward 5'-TTGCCTACCATCGTGACATCTCCA-3'  reverse 5'-CACCAGACAGAAGGACCAGAGT-3' | [3] |
| miR-122 | forward 5’-TAACACCTTCGTGGCTACAGA-3’  reverse 5’-ATATCAGATGAACCTTCTTGCTC-3’ | [4,5] |
| miR-151 | forward 5’-AGCTGAGCCTGGTGCTAGTCA-3’  reverse 5’-CCTGGGTGACTCTTCCTGTATCTA-3’ | [6] |
| miR-130a | forward 5'-GGAGAAGGAGTGAGGAGGCAGG-3'  reverse 5'-CCAGATGGGAGAGGAATTGCA-3' | [7] |
| miR-145 | forward 5'-GCAGAAGAGAACTCCAGC-3'  reverse 5'-TCATCCTGTGAGCCAGCCGAG-3' | [8] |
| miR-195 | forward 5'-GCCTCAAGAGAACAAAGTGGA-3'  reverse 5'-TTCGTGCTGTCTGCTTAACATT-3' | [9] |
| miR-199a-1 | forward 5'-CTTGGCTGCTCAGAGGTGCT-3'  reverse 5'-CGAATCTTCTATGCGAGGCT-3' | [10] |
| miR-199a-2 | forward 5'-GTGTGTCTCAGTCAGCG-3'  reverse 5-AACACTGGATATGAGATTCAA-3' | [11] |
| miR-214 | forward 5'-CAGGCTGATTGTATCTGTCTATGA-3'  reverse 5'-GGTTGTAGCTCTTGGTGTAGATG-3' | [12] |
| miR-221 | forward 5'-GCAGTAGGCAGTTGTGTTGAA-3'  reverse 5'-GGTAGCATTGGTGAGACAGC-3' | [13,14] |
| miR-222 | forward 5'-GTCACTCAGTCAGTATCTGTTG-3'  reverse 5'-CCAATAATCTCTCTCAGGACACT-3' | [15] |
| miR-223 | forward 5’-TCACATCTCCCAGGAAGATCTCA-3’  reverse 5’-TCCATTCGTCATATCCCATCTG-3’ | [16] |
| miR-224 | forward 5'-CTTGGCTGCTCAGAGGTGCT-3'  reverse 5'-CGAATCTTCTATGCGAGGC-3' | [17] |
| miR-301 | forward 5'-GCTTCAACCGATGCAAGATG-3'  reverse 5'-CAACATTATAGTTCTCAGCAACAA-3' | [11] |

**References**

1. Zhu XM, Wu LJ, Xu J, Yang R, Wu FS (2011) Let-7c microRNA expression and clinical significance in hepatocellular carcinoma. J Int Med Res 39: 2323-2329.

2. Liu WH, Yeh SH, Lu CC, Yu SL, Chen HY, et al. (2009) MicroRNA-18a prevents estrogen receptor-alpha expression, promoting proliferation of hepatocellular carcinoma cells. Gastroenterology 136: 683-693.

3. Meng F, Henson R, Wehbe-Janek H, Ghoshal K, Jacob ST, et al. (2007) MicroRNA-21 regulates expression of the PTEN tumor suppressor gene in human hepatocellular cancer. Gastroenterology 133: 647-658.

4. Hsu SH, Wang B, Kota J, Yu J, Costinean S, et al. (2012) Essential metabolic, anti-inflammatory, and anti-tumorigenic functions of miR-122 in liver. J Clin Invest 122: 2871-2883.

5. Tsai WC, Hsu SD, Hsu CS, Lai TC, Chen SJ, et al. (2012) MicroRNA-122 plays a critical role in liver homeostasis and hepatocarcinogenesis. J Clin Invest 122: 2884-2897.

6. Ding J, Huang S, Wu S, Zhao Y, Liang L, et al. (2010) Gain of miR-151 on chromosome 8q24.3 facilitates tumour cell migration and spreading through downregulating RhoGDIA. Nat Cell Biol 12: 390-399.

7. Xu N, Shen C, Luo Y, Xia L, Xue F, et al. (2012) Upregulated miR-130a increases drug resistance by regulating RUNX3 and Wnt signaling in cisplatin-treated HCC cell. Biochem Biophys Res Commun 425: 468-472.

8. Law PT, Ching AK, Chan AW, Wong QW, Wong CK, et al. (2012) MiR-145 modulates multiple components of the insulin-like growth factor pathway in hepatocellular carcinoma. Carcinogenesis 33: 1134-1141.

9. Ding J, Huang S, Wang Y, Tian Q, Zha R, et al. (2013) Genome-wide screening revealed that miR-195 targets the TNF-alpha/NF-kappaB pathway by downregulating IKKalpha and TAB3 in hepatocellular carcinoma. Hepatology.

10. Fornari F, Milazzo M, Chieco P, Negrini M, Calin GA, et al. (2010) MiR-199a-3p regulates mTOR and c-Met to influence the doxorubicin sensitivity of human hepatocarcinoma cells. Cancer Res 70: 5184-5193.

11. Jiang J, Gusev Y, Aderca I, Mettler TA, Nagorney DM, et al. (2008) Association of MicroRNA expression in hepatocellular carcinomas with hepatitis infection, cirrhosis, and patient survival. Clin Cancer Res 14: 419-427.

12. Wang X, Chen J, Li F, Lin Y, Zhang X, et al. (2012) MiR-214 inhibits cell growth in hepatocellular carcinoma through suppression of beta-catenin. Biochem Biophys Res Commun 428: 525-531.

13. Yuan Q, Loya K, Rani B, Mobus S, Balakrishnan A, et al. (2013) MicroRNA-221 overexpression accelerates hepatocyte proliferation during liver regeneration. Hepatology 57: 299-310.

14. Fornari F, Gramantieri L, Ferracin M, Veronese A, Sabbioni S, et al. (2008) MiR-221 controls CDKN1C/p57 and CDKN1B/p27 expression in human hepatocellular carcinoma. Oncogene 27: 5651-5661.

15. Wong QW, Ching AK, Chan AW, Choy KW, To KF, et al. (2010) MiR-222 overexpression confers cell migratory advantages in hepatocellular carcinoma through enhancing AKT signaling. Clin Cancer Res 16: 867-875.

16. Wong QW, Lung RW, Law PT, Lai PB, Chan KY, et al. (2008) MicroRNA-223 is commonly repressed in hepatocellular carcinoma and potentiates expression of Stathmin1. Gastroenterology 135: 257-269.

17. Wang Y, Lee AT, Ma JZ, Wang J, Ren J, et al. (2008) Profiling microRNA expression in hepatocellular carcinoma reveals microRNA-224 up-regulation and apoptosis inhibitor-5 as a microRNA-224-specific target. J Biol Chem 283: 13205-13215.
