# Supplementary material for: ADAR2-Mediated Editing of miR-214 and miR-122 Precursor and Antisense RNA Transcripts in Liver Cancers
Source: PLoS One. 2013 Dec 27;8(12):e81922. doi: 10.1371/journal.pone.0081922 (PMC3873926; doi:10.1371/journal.pone.0081922)
Supplement: Table S2 — The relative signal scores for the HRM analysis of the precursors of 16 HCC related miRNAs from Huh-7 cells infected with specific lenti-ADARs. (DOCX) [file pone.0081922.s002.docx]

**Table S2. The relative signal scores for the HRM analysis of the precursors of 16 HCC related miRNAs from Huh-7 cells infected with specific lenti-ADARs.**

†, the relative signal scores were calculated by HRM Gene Scanning Software, by comparing with the “no editing” standards.

*, the miRNAs showing HRM pattern significantly different from the “no editing” standards (> cut-off value of 5).
